# Aberrant functional brain connectome in people with antisocial personality disorder

**DOI:** 10.1038/srep26209

**Published:** 2016-06-03

**Authors:** Yan Tang, Jun Long, Wei Wang, Jian Liao, Hua Xie, Guihu Zhao, Hao Zhang

**Affiliations:** 1 Department of Neurology, Xiangya Hospital, Central South University, Changsha, Hunan 410078, China; 2 Biomedical Engineering Laboratory, School of Geosciences and Info-physics, Central South University, Changsha, Hunan 410083, China; 3 School of Information Science and Engineering, Central South University, Changsha, Hunan 410083, China; 4 Department of Electrical and Computer Engineering, Texas Tech University, Lubbock, TX 79409, USA

## Abstract

Antisocial personality disorder (ASPD) is characterised by a disregard for social obligations and callous unconcern for the feelings of others. Studies have demonstrated that ASPD is associated with abnormalities in brain regions and aberrant functional connectivity. In this paper, topological organisation was examined in resting-state fMRI data obtained from 32 ASPD patients and 32 non-ASPD controls. The frequency-dependent functional networks were constructed using wavelet-based correlations over 90 brain regions. The topology of the functional networks of ASPD subjects was analysed via graph theoretical analysis. Furthermore, the abnormal functional connectivity was determined with a network-based statistic (NBS) approach. Our results revealed that, compared with the controls, the ASPD patients exhibited altered topological configuration of the functional connectome in the frequency interval of 0.016–0.031 Hz, as indicated by the increased clustering coefficient and decreased betweenness centrality in the medial superior frontal gyrus, precentral gyrus, Rolandic operculum, superior parietal gyrus, angular gyrus, and middle temporal pole. In addition, the ASPD patients showed increased functional connectivity mainly located in the default-mode network. The present study reveals an aberrant topological organisation of the functional brain network in individuals with ASPD. Our findings provide novel insight into the neuropathological mechanisms of ASPD.

People with antisocial personality disorder (ASPD) often display symptoms of callousness, social anhedonia, irritability and impulsivity^1^. Previous neuroimaging studies have suggested the behavioural abnormalities of ASPD might have a neurobiological basis potentially involving structural, metabolic and functional abnormalities^2^. Mounting evidence indicates that these abnormalities are related to deficits in the prefrontal cortex, temporal cortex, insula, angular gyrus, parahippocampus, and anterior/posterior cingulate gyrus. In our previous studies, we investigated ASPD-related changes in the regional homogeneity (ReHo) by using resting-state functional MRI (R-fMRI) data, and we found that the ASPD individuals show reduced ReHo in the middle frontal gyrus and elevated ReHo in the inferior temporal gyrus^3^. In another functional connectivity study^4^, we determined that ASPD-associated changes occur primarily within and across the default-mode network (DMN). These findings provide new evidence for a disrupted resting-state functional connectivity in people with ASPD, as well as disrupted functional integrity among widely distributed brain regions. However, it remains unknown whether an aberrant topological organisation and abnormal assemblages exist within the connectivity networks of ASPD.

Graph theoretical analysis is a powerful tool for exploring the topological architecture of brain networks^5^ and the full connectivity of networks at a system level. Bullmore *et al*.^6^ have demonstrated that the functional networks directly supporting the neurophysiological dynamics of the brain show features of complex networks, such as small-world topology. Moreover, recent studies have indicated that several brain diseases are associated with an aberrant topological organisation pattern in the functional connectome. Such findings have deepened understanding of the human connectome in diseases including Alzheimer’s disease, schizophrenia, anxiety disorders, and depressive disorders. Hence, to better understand the topological organisation of ASPD, graph theory analysis was applied to explore the functional connectome in ASPD.

Some researchers have observed neuronal oscillations that are linearly distributed on a natural logarithmic scale, as well as distinct oscillators that maintain specific properties and physiological functions within specific frequency bands^7^. Accordingly, wavelet-based frequency band analysis has been proposed to reveal the pathophysiology of diseases^8^. These studies have suggested that the pattern of intrinsic brain activity might be sensitive to specific frequency bands. Therefore, it is necessary to identify these unique frequency bands to further understand the patterns of functional connectivity in people with ASPD.

In this study, we collected R-fMRI data to investigate the changes in the functional connectome in different frequency bands in individuals with ASPD. Our study focused on the topological architecture, specifically the global and local topological organisation, of the functional brain network in people with ASPD.

## Results

### Demographic and psychometric information

All subjects were male, and there were no significant differences between the two groups in age, educational level or IQ (p > 0.05). Notably, the score of the antisocial scale, determined by the PDQ (Personality Diagnostic Questionnaire), was significantly higher in the ASPD group (p < 0.0001; see Table 1). Table 1 presents the data describing the mental disorders in the ASPD group, which included hypochondriasis, depression, hysteria, psychopathic deviate, masculinity-femininity, paranoia, psychasthenia, schizophrenia, mania and social introversion.

### Frequency-specific alterations in the wavelet correlation matrix

**Figure 1:**
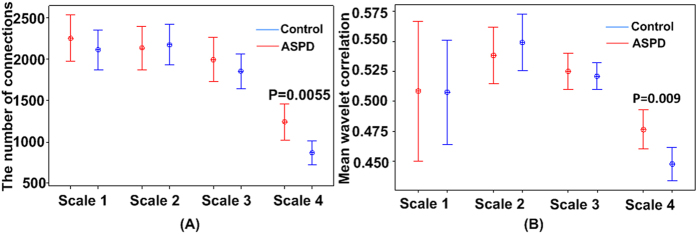
Between-group differences in L-AAL (n = 90). The left panel demonstrates the between-group differences in the number of connections. The right panel demonstrates the between-group differences in terms of the mean correlation connectivity. ASPD: Antisocial Personality Disorder; AAL: Anatomical Automatic Labelling.

The networks of individuals with ASPD had a significantly higher number of links (p = 0.0055) and a higher mean wavelet coefficient (p = 0.009) in wavelet scale four (0.016–0.031 Hz) (Fig. 1). No significant between-group differences were found in the other frequency bands. According to the binary networks definition (see Network Construction), more links in wavelet scale four in the ASPD group indicated that the positive correlations of connections were higher in subjects with ASPD. Hence the subsequent network topological analyses focused only on wavelet scale four and changes in its topological parameters. He *et al*.^9^ have demonstrated that the networks produced with the same p value threshold can capture the characteristics and organisational changes in the functional network. In some reports, e.g., Liao *et al*.^10^, He *et al*.^11^, and Cao *et al*.^12^, the topological parameters have been analysed without normalisation despite the different number of connections reported between the disease and control groups. Thus, normalisation was not used in the subsequent network topological analyses.

### Topological organisation of the functional connectome

Both ASPD and control subjects demonstrated typical features of small-worldness topology, as indicated by γ > 1 and λ ≈ 1. However, the ASPD group showed a significant decrease in γ (p = 0.0037) and σ (p = 0.0017). Additional comparisons revealed a significantly increased E_glob_ (p = 0.016), E_loc_ (p = 0.017) and C_p_ (p = 0.007) but a decreased L_p_ (p = 0.0497) in the functional brain networks of ASPD versus control subjects (Table 2, in Supplementary Fig. S1).

### Between-group differences in betweenness centrality

**Figure 2:**
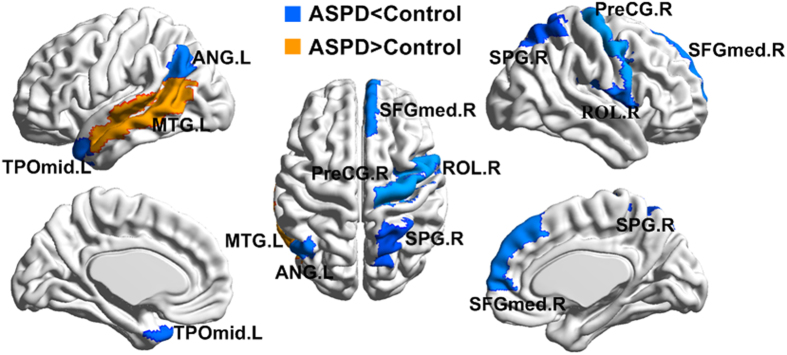
Brain regions showing the abnormal nodal betweenness centrality in ASPD compared with controls (p < (1/N) = 0.011). ANG.L: left angular gyrus; MTG.L: left middle temporal gyrus; TPOmid.L: left middle temporal pole; SFGmed.R: right medial superior frontal gyrus; PreCG.R: right precental gyrus; ROL.R: right Rolandic operculum; SPG.R: right superior parietal gyrus; ASPD: Antisocial Personality Disorder.

The significance of the differences between groups in these metrics and at each region was tested using two-sample t tests. The statistical significance was corrected for multiple comparisons using a false-positive adjustment^13^,^14^ of p < (1/N) = 0.011, where N = 90 corresponds to the number of comparisons given various exploratory analyses at nodal measures of topological attributes. The betweenness centralities were significantly different between ASPD and control subjects in several brain regions. A decreased betweenness centrality was observed in the right Rolandic operculum, right precentral gyrus, right medial superior frontal gyrus, right superior parietal gyrus, left angular gyrus and left middle temporal pole, whereas an increased betweenness centrality was found only in the left middle temporal gyrus (Fig. 2 and Supplementary Table S1).

**Figure 3:**
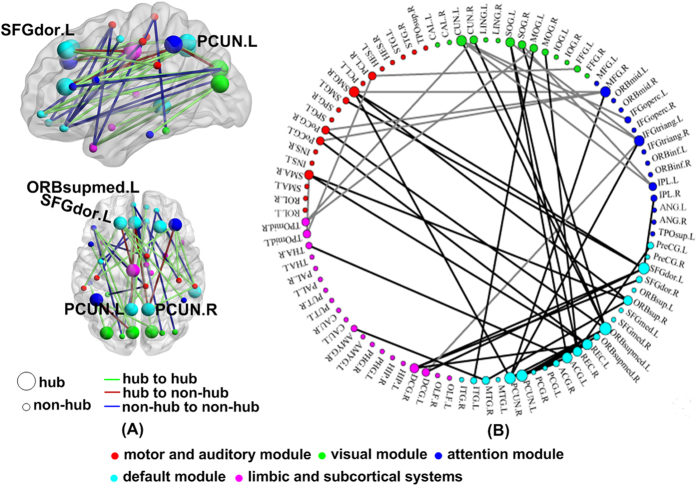
ASPD subjects showed aberrant functional networks according to the network-based statistical (NBS) analysis method and under a liberal primary threshold of (p < 5 × 10^−4^). Regions are colour-coded by category. The line represents the functional connection. (**A**) A single network of 47 connections is presented. (**B**) Ninety brain regions are displayed, containing five modules in a circle graph. The abnormal functional connection is demonstrated with a line. The greater the number of connections in a region, the larger the region is. ASPD: Antisocial Personality Disorder.

**Figure 4:**
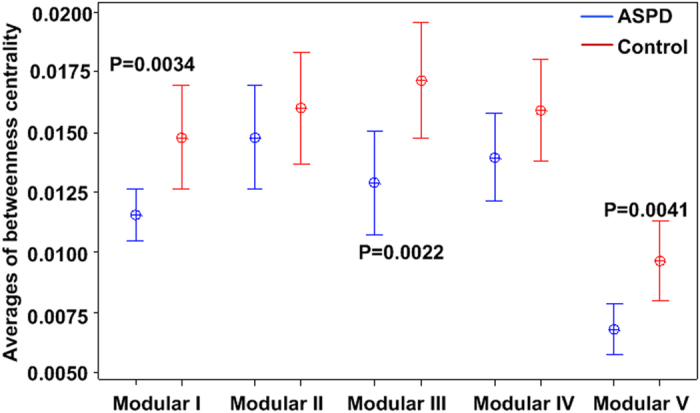
The between-group differences (p < (1/N) = 0.011) in the averages betweenness centralities. Significant differences were found in Module III (p = 0.0022), Module I (p = 0.0034) and Module V (p = 0.0041). Module I: somatosensory and auditory module; Module II: visual module; Module III: attention module; Module IV: default-mode network module; Module V: limbic/paralimbic and subcortical systems. ASPD: Antisocial Personality Disorder.

A modular architecture was derived from a resting-state functional connectome modularity study^15^ (Supplementary Table S2 and Fig. 3(B)). The modular architecture included the following five modules: Module I, the somatosensory and auditory module; Module II, the visual module; Module III, the attention module; Module IV, DMN module; and Module V, the limbic/paralimbic and subcortical systems. Between-group differences were further identified in the average betweenness centrality within modules. Bonferroni correction with a threshold of p < 0.05/5 = 0.01 was used to test for significant differences in the betweenness centrality for these modules. Compared with that in the control subjects, the average betweenness centrality was significantly lower in the ASPD subjects in the somatosensory and auditory modules (p = 0.0034), limbic and subcortical systems (p = 0.0041) and attention module (p = 0.0022) (Fig. 4).

Functional hubs were identified using the normalised average betweenness centrality in the ASPD and control groups. The ASPD and control groups had highly similar hubs in terms of the distribution, i.e., the right dorsolateral superior frontal gyrus, left supramarginal and angular inferior parietal gyrus, bilateral precuneus, bilateral middle frontal gyrus, bilateral superior temporal gyrus, right middle temporal gyrus, right cuneus, left middle cingulate gyrus, and right superior temporal pole (Supplementary Fig. S2), results consistent with findings from previous studies^11^. Further analysis identified the hubs unique in ASPD individuals, i.e., the left dorsolateral superior frontal gyrus, left anterior cingulate gyrus, left cuneus, left middle occipital gyrus and left middle temporal gyrus. Similarly, several other regions were identified as hubs only in the control subjects, i.e., the right parahippocampal gyrus, left lingual gyrus, left inferior parietal lobule, left superior temporal pole and right inferior temporal gyrus.

### Aberrant functional network connectivity

A network-based statistical (NBS) analysis revealed a significant increase in functional connections in ASPD versus control subjects. Under the primary threshold (p < 5 × 10^−4^), a single network with 47 links among widely distributed brain structures was identified in the ASPD group (p = 0.001, corrected) (Fig. 3). Intriguingly, most of these connections (33 of 47) were long-distance connections (Euclidean distance > 75 mm)^11^. In the subsequent analyses, these abnormal functional connections were divided into three groups: links between the hubs, between the hub and non-hub, and between the non-hubs. We found the aberrant connections were predominantly links between the hubs and non-hubs (hub to hub: 5; hub to non-hub: 33; non-hub to non-hub: 14). Additionally, these abnormal connections were mostly considered to be intermodule connections between the DMN and the other networks (Fig. 3B).

### Correlation between topological metrics and clinical variables

**Figure 5:**
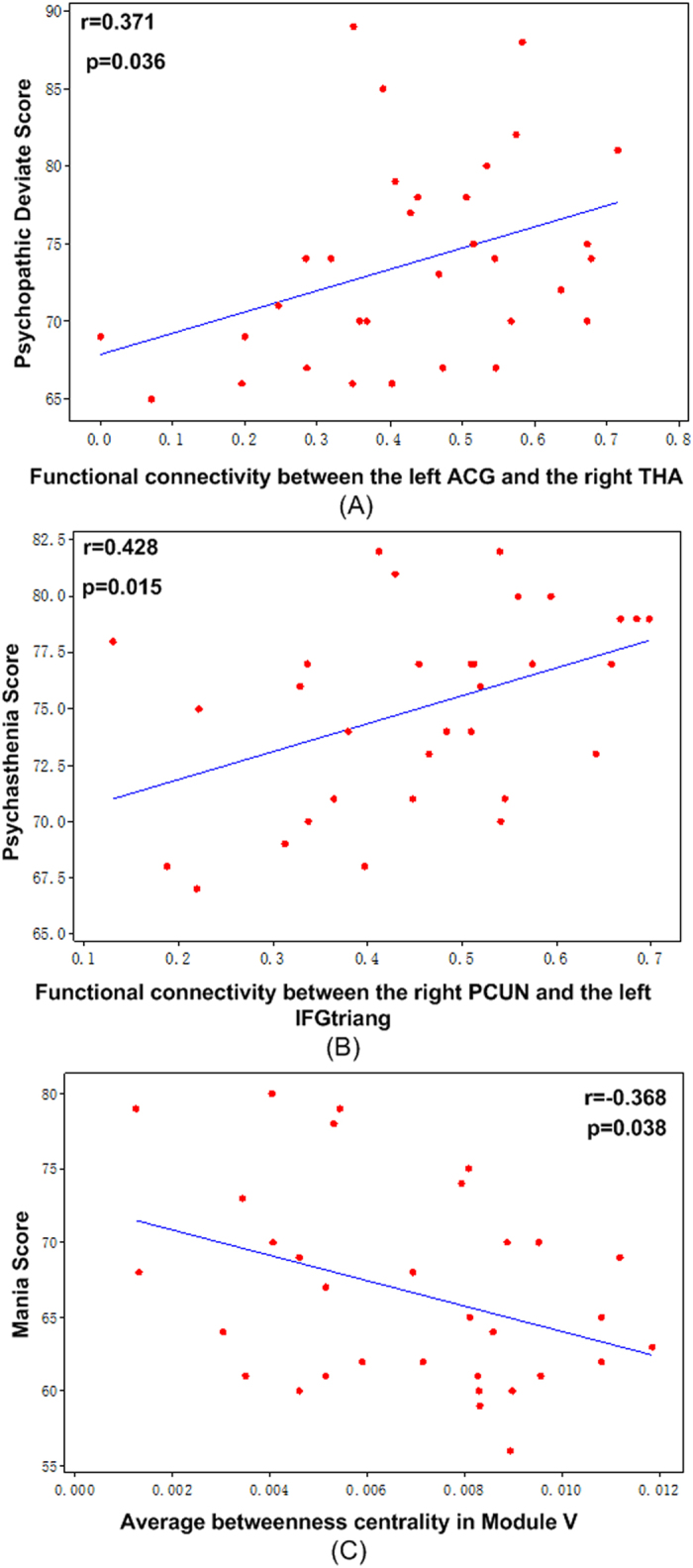
Correlations between the topological metrics and clinical variables. (**A**) The functional connectivity between the left anterior cingulate gyrus and the right thalamus was positively (p < 0.05) correlated with the psychopathic deviate score. (**B**) The functional connectivity between the right precuneus and the left inferior frontal gyrus (triangular part) was positively (p < 0.05) correlated with the psychasthenia score. (**C**)The average betweenness centrality in Module V showed a negative (p < 0.05) correlation with the mania score. ACG: anterior cingulate gyri; THA: thalamus; PCUN: precuneus; IFGtriang: inferior frontal gyrus (triangular part); Module V: the limbic/paralimbic and subcortical systems.

None of the global topological metrics were significantly correlated with the scores for all mental disorders, which included hypochondriasis, depression, hysteria, psychopathic deviate, masculinity-femininity, paranoia, psychasthenia, schizophrenia, mania, and social introversion. The functional connectivity between the right precuneus and the left inferior frontal gyrus was positively correlated with the psychasthenia score (p < 0.05). The functional connectivity between the left anterior cingulate gyrus and the right thalamus was positively (p < 0.05) correlated with the psychopathic deviate score. The average betweenness centrality in the limbic and subcortical systems showed negative (p < 0.05) correlations with the mania score. (Fig. 5).

### Reproducibility of the findings in the wavelet-derived network metrics

First, the regional parcellation effects were evaluated. When using L-Crad (n = 200) atlas^16^, some parameters, such as the E_glob_, E_loc_ and C_p_, increased significantly, whereas the small-world index γ and L_p_ were significantly reduced in the ASPD versus control subjects. Similar results were found using automated anatomical labelling (AAL) (n = 90) (Supplementary Table S3). The same trend of an increase in the topological properties in subjects with ASPD was also observed when using the H-1024 (n = 1024) parcellation scheme^17^, although this result was not statically significant at the p < 0.05 level. Yet, the distributions of the abnormal nodal betweenness centrality, as indicated by the L-Crad atlas (n = 200) and the H-1024 parcellation scheme (n = 1024), were not consistent with the result from the AAL (n = 90), thus suggesting the influence of various parcellation schemes on the regional properties of the functional brain network^6^.

Second, as shown in Table 2, significant differences in network properties were identified between the ASPD and control groups in weighted networks by the nonparametric permutation tests (see Methods), and the same results were obtained in the binary network. The C_*p*_, E_glob_ and E_loc_ of the binary and weighted networks were significantly higher in the ASPD group; the *L*_p_, γ, σ of the binary and weighted networks were significantly smaller in the ASPD group. No significant difference in λ was observed between the two groups. In most cases, the weighted networks showed a much smaller p value than the binary networks, suggesting that the differences in network properties between these two groups were more significant in the weighted networks^18^.

Next, although there was no significant difference in the six head motion parameters between the ASPD and control subjects (p > 0.05), the effect of head motion was explored by applying the ‘scrubbing’ method to the pre-processed images^19^. The E_glob_, E_loc_, and C_*p*_ values increased significantly, and the normalised clustering coefficient γ and small-world index σ decreased significantly in the ASPD versus control subjects (Supplementary Table S4). There were few changes in the distributions of the abnormal nodal betweenness centrality.

## Discussion

In this study, the topological organisation of the functional brain networks in subjects with ASPD was examined. The abnormal organisation of the functional connectome in the brains of ASPD subjects was predominantly in wavelet scale four (0.016–0.031 Hz), which is part of the frequency range (0.01–0.073 Hz) for grey matter-related oscillations^20^. This abnormality in the subjects with ASPD can be characterised as follows: (i) an increase in the clustering coefficient; (ii) abnormal nodal betweenness in the frontal lobe, parietal lobe, and temporal lobe; (iii) decreased betweenness centralities in the somatosensory and auditory modules, attention module and limbic and subcortical systems; and (iv) an aberrant functional connectivity between the DMN and the other networks.

In this paper, a significantly higher total number of functional connections were observed in wavelet scale four (0.016–0.031 Hz) in subjects with ASPD. Previous studies have suggested that neuronal oscillations are linearly distributed on the natural logarithmic scale, and this regularity indicates that different frequency bands result from different oscillators with different biological properties and functions^21^. Although the exact mechanisms remain poorly understood, various brain diseases, such as amnestic mild cognitive impairment^7^, and major depressive disorder^22^, have shown disrupted topological architectures in specific frequency bands. Recent work has highlighted that nicotine increases the efficiency of the brain functional network in the frequency interval between 0.01 and 0.03 Hz^23^. These previous findings along with our present results highlight the clinical significance of brain oscillations between 0.016 and 0.031 Hz.

Van Wijk *et al*.^24^ have demonstrated that for networks with the same number of nodes, the normalised characteristic path length λ, and the clustering coefficient C_p_ better reflect the small-worldness of empirical networks with a different number of connections than the small-world index *σ*. Hence, a change in the clustering coefficient was considered in our work. A higher mean clustering coefficient represents greater local efficiency of information transferred in the brain network^25^. For consolidation of fear learning, the mean clustering coefficient increases in local limbic circuits, indicating enhanced regional processing efficiency^26^. Moreover, in R-fMRI, schizophrenia patients tend to have higher mean scores using the positive and negative syndrome scale (PANSS), which have been found to be positively correlated with the clustering coefficient during the resting state^27^. A recent network analysis has demonstrated that people with higher brain wave rhythms and increased clustering coefficients in specific frequency ranges tend to achieve higher scores in some cognitive tests^28^. A very similar resting EEG activity study^29^ has reported that individuals in an ASPD group show greater beta energy in some regions, as compared with the control group. Therefore, considering that ASPD is often associated with characteristics such as impulsivity, irritability, mania, and aggressive behaviour^1^, we hypothesised that people with these ASPD-related characteristics might exhibit higher activity in some brain areas within specific frequency ranges, which would be reflected by an increased clustering coefficient.

Unlike previous studies of structural, metabolic and functional abnormalities, the abnormal nodal betweenness in ASPD subjects was examined in our study to evaluate the influence of a node on the information flow between the remaining nodes in a network. Nevertheless, the nodal betweenness was significantly positively correlated with blood flow and metabolism^30^. Our observations extended the current understanding of the neuroanatomical features of individuals with ASPD. All of the abnormal ROIs were related to the symptoms of depersonalisation (Supplementary Discussion). The abnormal nodal betweenness may suggest high impulsivity, lack of conscience, and cold-bloodedness in ASPD.

Additionally, to characterise the local properties of the network and to assess the potential effects of the nodes on the network dynamics, the average within-module betweenness centrality was investigated to reveal information flow patterns in brain networks. The average within-module betweenness centrality is high when many nodes in the module are on the network’s shortest paths, which means many nodes in the module act as the “bridges” along pathways with the strongest coupling. Therefore, the analysis of the changes in the average betweenness centrality in a given module reflects the role of the module in the transfer of information through the network. In our study, some modules in ASPD subjects, such as the somatosensory and auditory modules, attention module and limbic and subcortical systems, showed a decreased betweenness centrality. These alterations corresponded to the changes in the functional organisation of the brain network in ASPD subjects. Pham TH^31^ has provided support for the hypothesis of selective attention and specific executive function deficits among subjects with ASPD through a series of psychological scales. Specifically, previous studies have found that ASPD patients have a deficiency in the orbitofrontal cortex and dorsolateral prefrontal cortex^32^,^33^, which constitutes the attention module. These lesions may be the main cause of disinhibited, impulsive and unconcerned behaviours in subjects with ASPD^34^,^35^, a neurological syndrome significantly defined as an “acquired sociopathy”^36^. It has been argued that the deficiency in the attention module may lead to poor cognitive control and reduced attention in individuals with antisocial personalities. Furthermore, many personality disorders involve a dysfunction of subcortical structures or the limbic or paralimbic cortex^37^,^38^. Starkstein *et al*.^39^ have observed hypometabolism in subcortical structures with increased irritability. In this study, it was found that the decreased average betweenness centrality in the limbic or paralimbic cortex was associated with increased erratic emotions in ASPD subjects. Therefore, it is argued that the deficient information exchange in the limbic and subcortical systems may be related to ASPD individuals being irritable, capricious and impulsive.

However, via the NBS analysis, most of the connections with significantly increased functional connectivity were long-distance connections and between the hub and non-hub nodes. ASPD was associated with a reorganisation of the hub structure (Supplemental Fig. S2), which has been suggested to form during childhood in ASPD patients^40^,^41^. The fine-tuning of connections between functional hubs and non-hub regions may influence and support developmental improvements in cognition^40^. Specifically, theses abnormal links occurred predominately between the DMN and other functional modules, consistently with results from our previously study^4^. The DMN is related to emotional regulation, future planning, self-inspection^42^, and mind-wandering^43^. Moreover, the functional connectivity between the right precuneus and the left inferior frontal gyrus (triangular part) was positively correlated with the psychasthenia score, and the functional connectivity between the left anterior cingulate gyrus and the right thalamus was positively correlated with the psychopathic deviate score. As part of the DMN and the “hub”, the precuneus was involved in self-consciousness, and the anterior cingulate gyrus was in charge of conflict monitoring. Therefore, these increased connections between the DMN and other networks may be interpreted as facilitating transmission of conflicted emotions and hampering the ability to control impulses, which manifests as irritability and impulsivity in subjects with ASPD.

Together, our results suggest that an abnormal functional network, such as increased connections in the DMN and decreased betweenness centrality in the limbic systems and attention module, might lead to inflated self-appraisal, disregard for the rights of others and a lack of empathy, which are characteristics of subjects with ASPD.

Several issues need to be addressed. Mounting evidence suggests that functional connectivity correlates with structural (anatomical) connectivity patterns at an aggregate level^44^,^45^, and structural networks help to understand the fundamental architecture of inter-regional connections^6^. Therefore, more attention should be paid to the relationship between the structural and functional connectomics in subjects with ASPD in future studies.

To our knowledge, our work is the first to explore frequency-dependent topological alterations in people with ASPD. Compared with the control group of subjects matched for age, gender and education level, the group containing individuals with ASPD showed both global and regional topological alterations in the specific and most salient frequency range between 0.016 and 0.031 Hz. Our findings not only provide novel insight into the neuropathological mechanisms of ASPD but also highlight the importance of frequency-dependent information when investigating ASPD.

## Materials and Methods

### Subjects

In total, 320 volunteers were recruited for the experiment from the School for Youth Offenders of Hunan Province. All volunteers had received reformatory education in this school because they had committed misdemeanours. All young offenders had regular daily school hours. “Enclosed-style” management was implemented in this special school. In this study, participants selected for the fMRI experiments were accompanied by three teachers as they underwent fMRI scanning. All subjects were of legal age to give consent (age > 18) at the time of the experiment, but they were under the legal age when they first began attending the school.

First, all volunteers were tested in groups by an experienced psychological testing professional using the Personality Diagnostic Questionnaire-4+ (PDQ-4+). Among the 320 subjects, 122 received an ASPD score that was equal to or greater than four, whereas the other subjects were excluded from the study. The remaining 122 subjects received further testing with the Personality Disorder Interview (PDI-IV)^46^, which was conducted by two senior psychiatrists. The inter-rater consistency of the score of the PDI-IV was 0.897. From these subjects, 45 were selected based on a diagnosis of ASPD and because they did not have a history or current diagnosis of a serious mental disorder (e.g., depression or anxiety neurosis). However, the data for 13 ASPD of these 45 subjects were removed because they had movement-related artefacts that were identified in post-scanning (see Data Preprocessing). Therefore, the final experimental population for this study consisted of 32 ASPD individuals. Meanwhile, to further investigate mental disorders in individuals with ASPD, the 32 ASPD subjects completed handwritten versions of the MMPI-2 (Minnesota Multiphasic Personality Inventory) questionnaires^47^. The reliability, cross-instrument validity and factor structure of Chinese adaptations of the PDQ-4 and PDI- IV in psychiatric patients have been evaluated by Yang *et al*.^48^, who have suggested that the psychometric properties of these two instruments are comparable across different cultures.

The remaining 198 volunteers who did not meet the ASPD criteria of the PDQ-4+ were also tested with the PDI-IV. Fifty participants with no history or current diagnosis of a serious mental disorder, (e.g., depression or anxiety neurosis), were selected as the non-ASPD controls. Eighteen control subjects also were removed because they exhibited movement-related artefacts that were identified in post-processing (see Data Preprocessing), and 32 subjects remained as the control group.

All participants were male and native Chinese speakers, and they had no access to alcohol or illicit drugs for at least six months prior to the brain scan. Overall, the participants were strongly right-handed, as judged by the Lateral Dominance Test^49^ and had normal or corrected-to-normal vision. In addition, a short version of the Wechsler Adult Intelligence-Scale Revised in China (WAIS-RC)^50^, including four combinations of information, similarities, picture completion and block design subtests, was used to measure the intelligence quotient (IQ) of all volunteers.

After receiving a detailed description of the study, all volunteers provided written informed consent. All the potential participants made the choice based on their free will, and those who declined to participate or did not participate for other reasons were treated in the same way as those who participated. Written informed consent, was obtained from each subject before the experiment. All experimental protocols were approved by the research ethical committee of Central South University of China. The methods were carried out in accordance with the approved guidelines.

### Image acquisition

Each participant underwent an R-fMRI scan using a Siemens Avanto 1.5-T system at the Xiangya Hospital of Central South University, Changsha, with the following parameters: TR = 2000 ms, TE = 50 ms, slice thickness = 5 mm, flip angle = 90°, matrix = 64 × 64, slices number = 23, and voxel size = 3.75 × 3.75 × 6.25 mm. For each participant, 150 image volumes were acquired over five minutes. During data acquisition, all subjects were instructed to keep their eyes closed, relax their minds and move as little as possible.

The structural data were acquired by T1-weighted brain MRI scans using a standard sagittal 3D MP-RAGE sequence (TR = 2400 ms, TE = 3.61 ms, flip angle = 8°, FOV = 240 × 240 mm, slice thickness = 1.2 mm, slices number = 160, voxel size = 1 × 1 × 1 mm).

### Data preprocessing

The fMRI images were pre-processed using the SPM8 package (www.fil.ion.ucl.ac.uk/spm; Wellcome Trust Centre for Neuroimaging, University College London, United Kingdom) and Data Processing Assistant for Resting-State fMRI (DPARSF)^51^ (http://www.restfmri.net). For each subject, the first five volumes of the scanned data were discarded because of T1 equilibration effects, leaving 145 images available for the analysis. Subsequent data preprocessing steps included within-subject slice timing, head motion correction and between-subject spatial normalisation to the standard Montreal Neurological Institute (MNI) template with a resampled voxel size of 3 × 3 × 3 mm.

After the head motion correction procedure, the magnitude of head motion at each time point was determined for each subject by using six parameters (three for shift and three for rotation). Subjects with head motion exceeding a maximum displacement of 1 mm at any axis and the angular motion of 1° in any direction were excluded from the further analysis, based on which 13 ASPD subjects and 18 control subjects were excluded, leaving 32 ASPD subjects and 32 control subjects. The average head motion parameters of shift and rotation were then calculated, but they showed no significant differences in head motion between the two groups (p > 0.05). Detrending and temporal high-pass filtering (HPF cutoff frequency = 0.01 Hz) were applied.

### Network construction

Given the concerns of the accuracy of the result of the affine linear registration on the cerebellum^52^, a subset of 90 non-cerebellar regions-of-interest (ROIs) as the nodes in brain networks (45 for each hemisphere, according to AAL atlas^53^) were used to investigate abnormal functions in ASPD individuals.

The mean time series were obtained for each subject by averaging the fMRI time series across all voxels in each of the 90 regions. Six head motion parameters were regressed out from the extracted time series as nuisance covariates. The global signal was kept in our analysis to avoid the anti-correlations trend caused by removing global signal during the preprocessing of R-fMRI data^54^,^55^ and coincide with results from previous studies of wavelet-based functional brain network studies.

The maximal overlap discrete wavelet transform (MODWT) over the first four scales was applied to the mean time series of each ROI. The resulted frequency range of the k^th^ scale of the wavelet decomposition was [2^(−k−1)^/TR, 2^(−k)^/TR] corresponding to four scales (scale 1, 0.125–0.250 Hz; scale 2, 0.063–0.125 Hz; scale 3, 0.031–0.063 Hz; and scale 4, 0.016–0.031 Hz)^8^. The wavelet coefficient was used as a measure of functional connectivity^23^. Inter-regional pairwise Pearson correlations and their corresponding statistical significance levels (i.e., p values) were computed for each of the four scales, which led to four 90 × 90 correlation matrices for each subject. A detailed description can be found in Percival and Walden^8^ and is available in the waveslim package in the R language (www.cran.r-project.org).

The binary connection matrix can capture the underlying functional connectivity patterns of the human brain^11^ and facilitate understanding of complex systems, which was constructed from the correlation matrix in our initial study. The p value, instead of the correlation coefficient, was used as a threshold because it allows for a greater certainty of the inner-correlation between two ROIs^10^. He *et al*.^9^ have shown that the networks produced with the same p value threshold also capture the network characteristics and the organisational changes in the functional network.

Because the correlation analysis was performed over all 90 × 89/2 = 4005\combinations of ROIs, a multiple comparisons correction was needed to test the significance of these correlations. A strict Bonferroni correction was applied for multiple comparisons with the threshold equal to 0.05/4005 = 1.2484 × 10^−5^ to remove spurious correlations. Using this threshold (p < 1.2484 × 10^−5^), a symmetric binary connectivity matrix A_ij_ = [a_ij_] was constructed, which was either one (indicating that the functional correlation between two regions was considered to be statistically significant) or zero (indicating that the correlation was not statistically significant).


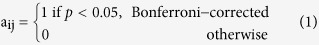


Given the ambiguous physiological significance of the negative correlation, these types of connectivity were removed from the subsequent analyses (that is, a_ij_ was 0 if the Pearson’s correlation coefficient was negative).

### Network analysis

All network analyses were performed using the Brain Connectivity Toolbox (BCT)^25^.

Four network metrics, including two small-world parameters (clustering coefficient C_*p*_, and characteristic path length L_*p*_) and two efficiency parameters (global efficiency *E*_*glob*_, and local efficiency *E*_*loc*_), were used to characterise the global topological organisation of brain networks. The definitions and descriptions of these four parameters can be found in Rubinov and Sporns^25^.

To estimate the small-world properties of the constructed functional networks, 1000 random graphs were generated via the random rewiring, which yielded random graphs with the same number of nodes, mean degree and degree distribution as the real network. 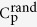 and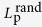 were defined as the mean clustering coefficient and the mean characteristic path length of 1000 matched random networks, respectively. Typically, a small-world network should meet the following criteria: normalised clustering coefficient 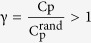, normalised characteristic path length 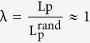 and small-worldness index 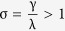.

The regional characteristics of the brain networks that measure a given node connecting with all other nodes may indicate the importance of specific brain areas in the network. To characterise the local properties of nodes, the betweenness centrality was adopted, which is defined as the fraction of all shortest paths in the network. It is important to note that such a definition is very different from a measure based on degree in which only the number of connections is taken into account. Thus, betweenness centrality is less sensitive to the eventual inflation induced by the community size^56^. Betweenness centrality B_i_, where *i* is defined as the fraction of all shortest paths in the network, is a relatively more sensitive measure than other metrics to identify central nodes^25^, which is calculated as follows:


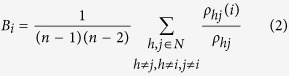


where *ρ*_*hj*_ is the number of shortest paths between node h and node *j*, and 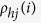 is the number of shortest paths between node h and node *j* that pass through node *i*. Therefore, betweenness centrality may be normalised to the range [0, 1].

To identify highly central nodes in a network, the betweenness centrality was further normalised as follows:


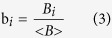


where <*B*> is the average betweenness centrality of the network. In this paper, b_*i*_ of the cortical region for each participant was examined and then averaged across the two groups. The ROIs with the large normalised betweenness centrality were identified as hubs (b_*i*_ > 1.5, i.e., a node with 1.5 times greater than the average betweenness centrality of the network)^57^. The altered hubs in the topological organisation were focused.

In addition, the mean betweenness centrality in different modules of the network was also investigated using a modular architecture which was derived from the resting-state functional connectome modularity study^15^, including the following five modules: Module I, the somatosensory and auditory module; Module II, the visual module; Module III, the attention module; Module IV, DMN module; and Module V, the limbic/paralimbic and subcortical systems. Between-group differences of the average betweenness centrality within modules were further identified.

### Statistical analysis

The significant differences in the topological attributes of the group (both global and nodal measures) were determined via nonparametric permutation tests. For each network metric, the mean values of the between-group differences were computed, which were randomly reallocated into two groups, and the mean differences between the two randomised groups were calculated. This randomisation procedure was repeated 10,000 times, and the 95th percentile points of the empirical distribution were used as the critical values for a one-tailed test of the null hypothesis with a probability of a type I error of 0.05.

An abnormal functional connectivity in ASPD individuals was identified using the NBS approach, the technical details of which are described in a previous publication^58^. In brief, a primary threshold was first used to determine a set of supra threshold links, within which any connected components, as well as their sizes (number of links), were then determined. To estimate the significance of each component, a corrected p value was calculated using the null distribution of the maximally connected component size, which was empirically derived from the aforementioned nonparametric permutation approach (10,000 permutations).

When significant between-group differences were observed in any network metrics, the relationships between these metrics and clinical variables (the scores of mental disorder in the MMPI-2 such as hypochondriasis, depression, hysteria, psychopathic deviate, masculinity-femininity, paranoia, psychasthenia, schizophrenia, mania, and social introversion) were further analysed. Multiple linear regression analyses with age as a confounding factor were performed.

### Reproducibility

Three effects were discussed to validate the reproducibility of our results. First, the effects of using different regional parcellation were evaluated, which might pose influence on the brain graph metrics, and two additional parcellation schemes, including the L-Crad (200 nodes)^16^ and H-1024 (1024 nodes)^17^, were employed to reanalyse the data and explore the reproducibility of our findings.

Second, the weights in functional networks representing respective magnitudes of correlational interactions were used to build a weighted network. Because the weighted characterisation usually focused on somewhat different and complementary aspects of network organisation, the parameters of topological organisations in the weighted networks were recalculated, and the difference was compared between the ASPD and control group.

Third, head motion effects were considered. Recently, several R-fMRI studies have reported the influence of head motion on the calculated functional connectivity^19^. To evaluate the substantial effects of head motion on our results, a “scrubbing” method was performed on the pre-processed images. For each subject, the pre-processed functional images with a frame-wise displacement between two time points greater than 0.5 mm were replaced by linear interpolating frames^19^. Then, the resulting “scrubbed” data were reanalysed.

## Additional Information

**How to cite this article**: Tang, Y. *et al*. Aberrant functional brain connectome in people with antisocial personality disorder. *Sci. Rep.* **6**, 26209; doi: 10.1038/srep26209 (2016).

## Supplementary Material

Supplementary Information

## Figures and Tables

**Table 1:** Demographic and psychometric information.

		ASPD (n = 32)	Controls (n = 32)	t	p
Age (year)		20.5 ± 1.37	21.67 ± 2.54	−0.92	0.36
Education level (year)		8.15 ± 1.54	8.73 ± 0.82	−0.82	0.45
Mean global IQ score		106.66 ± 12.90	106.84 ± 16.6	−0.50	0.51
Antisocial scale of PDQ		5.90 ± 0.4	2.06 ± 0.9	13.80	<0.0001
MMPI	Hypochondriasis	66.00 ± 6.62			
	Depression	49.35 ± 12.53			
	Hysteria	50.82 ± 8.50			
	Psychopathic deviate	73.78 ± 6.49			
	Masculinity-femininity	53.29 ± 8.35			
	Paranoia	62.17 ± 9.61			
	Psychasthenia	75.06 ± 4.30			
	Schizophrenia	74.26 ± 8.88			
	Mania	66.72 ± 6.61			
	Social introversion	53.17 ± 4.60			

Data are expressed as the mean ± SD. All subjects were right-handed men. The difference between the ASPD and control group was obtained by a two-sample t-test. ASPD: antisocial personality disorder; IQ: intelligence quotient; PDQ: personality diagnostic questionnaire.

**Table 2:** Comparisons of the global network metrics among the control and ASPD group in wavelet scale four.

Parcellation		E_glob_	E_loc_	L_p_	C_p_	λ	γ	σ
ASPD	B	0.52 ± 0.136	0.70 ± 0.145	2.19 ± 0.645	0.56 ± 0.130	1.12 ± 0.127	1.50 ± 0.494	1.32 ± 0.372
	W	0.29 ± 0.080	0.42 ± 0.072	0.833 ± 0.203	0.316 ± 0.072	1.07 ± 0.084	1.46 ± 0.534	1.34 ± 0.411
Control	B	0.45 ± 0.018	0.620 ± 0.135	2.42 ± 0.410	0.49 ± 0.117	1.14 ± 0.06	1.95 ± 0.799	1.7 ± 0.656
	W	0.25 ± 0.055	0.38 ± 0.066	0.90 ± 0.141	0.27 ± 0.062	1.09 ± 0.045	1.65 ± 0.550	1.50 ± 0.472
p value	B	0.016*	0.017*	0.0497*	0.007*	0.317	0.0037*	0.0017*
	W	0.0086*	0.03*	0.0448*	0.008*	0.231	0.0027*	0.0011*

Data are expressed as the mean ± SD. (*p < 0.05).

E_glob_: global efficiency; E_loc_: local efficiency; C_p_: clustering coefficient; L_p_: characteristic path length of a network; γ normalised clustering coefficient; λ: normalised characteristic path length; σ: small-worldness index; B: binary networks; W: weighted network.
