# Supplementary material for: Aberrant functional brain connectome in people with antisocial personality disorder
Source: Sci Rep. 2016 Jun 3;6:26209. doi: 10.1038/srep26209 (PMC4891727; doi:10.1038/srep26209)
Supplement: Supplementary Information [file srep26209-s1.pdf]

Aberrant functional brain connectome in people with antisocial personality disorder

Yan Tang[1,2], Jun Long[2*], Wei Wang[3], Jian Liao[3], Hua Xie[4], Guihu Zhao[2], and Hao Zhang[2]

1. Biomedical Engineering Laboratory, School of Geosciences and Info-physics, Central South University, Changsha, Hunan 410083, China

2. School of Information Science and Engineering, Central South University, Changsha, Hunan 410083, China

3. Department of Radiology, The Third Xiangya Hospital, Central South University, Changsha, Hunan 410083, China

4. Department of Electrical and Computer Engineering, Texas Tech University, Lubbock, TX 79409, USA

* Correspondence to jlong@csu.edu.cn

Table S1. Regions showing abnormal nodal betweenness centrality in ASPD subjects.

	Brain regions	Anatomical classification	p values of B_i (nodal betweenness)
ASPD < Control	Right superior parietal gyrus	Parietal	0.0034*
	Left middle temporal pole	Temporal	0.0042*
	Right Rolandic operculum	Frontal	0.0046*
	Left angular gyrus	Parietal	0.0049*
	Right medial superior frontal gyrus	Prefontal	0.0052*
	Right precental gyrus	Frontal	0.0063*
	Left parahippocampal gyrus	Temporal	0.0152
	Right parahippocampal gyrus	Temporal	0.0157
	Left inferior temporal gyrus	Temporal	0.0213
	Left inferior frontal gyrus, opercular part	Prefontal	0.0220
	Left olfactory cortex	Prefontal	0.0257
	Left middle frontal gyrus	Prefontal	0.0262
	Left lingual gyrus	Occipital	0.0275
	Right insula	Subcortical	0.0334
	Left temporal pole: superior temporal gyrus	Temporal	0.0356
	Right temporal pole: superior temporal gyrus	Temporal	0.0387
	Right inferior frontal gyrus, opercular part	Prefontal	0.0423
	Left superior frontal gyrus, orbital part	Prefontal	0.0428
	Right putamen	Subcortical	0.0447
ASPD > Control	Left middle temporal gyrus	Temporal	0.0064*
	Right paracentral lobule	Parietal	0.0266

*: $p < 1/90 = 0.011$.

Table S2. Module description[1]

Brain regions	Abbr.	Module	Regions	Abbr.	Module
Precentral gyrus	PreCG	Modular IV	Lingual gyrus	LING	Modular II
Superior frontal gyrus (dorsolateral)	SFGdor	Modular IV	Superior occipital gyrus	SOG	Modular II
Superior frontal gyrus (orbital part)	ORBsup	Modular IV	Middle occipital gyrus	MOG	Modular II
Middle frontal gyrus	MFG	Modular III	Inferior occipital gyrus	IOG	Modular II
Middle frontal gyrus (orbital part)	ORBmid	Modular III	Fusiform gyrus	FFG	Modular II
Inferior frontal gyrus (opercular part)	IFGoperc	Modular III	Postcentral gyrus	PoCG	Modular I
Inferior frontal gyrus (triangular part)	IFGtriang	Modular III	Superior parietal gyrus	SPG	Modular I
Inferior frontal gyrus (orbital part)	ORBinf	Modular III	Inferior parietal, but supramarginal and angular gyri	IPL	Modular III
Rolandic operculum	ROL	Modular I	Supramarginal gyrus	SMG	Modular I
Supplementary motor area	SMA	Modular I	Angular gyrus	ANG	Modular III
Olfactory cortex	OLF	Modular V	Precuneus	PCUN	Modular IV
Superior frontal gyrus (medial)	SFGmed	Modular IV	Paracentral lobule	PCL	Modular I
Superior frontal gyrus (medial orbital)	ORBsupmed	Modular IV	Caudate nucleus	CAU	Modular V
Rectus gyrus	REC	Modular IV	Lenticular nucleus, putamen	PUT	Modular V
Insula	INS	Modular I	Pallidum	PAL	Modular V
Anterior cingulate and paracingulate gyri	ACG	Modular IV	Thalamus	THA	Modular V
Median cingulate and paracingulate gyri	DCG	Modular V	Heschl gyrus	HES	Modular I
Posterior cingulate gyrus	PCG	Modular IV	Superior temporal gyrus	STG	Modular I
Hippocampus	HIP	Modular V	Temporal pole: superior temporal gyrus	TPOsup	Modular III
Parahippocampal gyrus	PHG	Modular V	Middle temporal gyrus	MTG	Modular IV
Amygdala	AMYG	Modular V	Temporal pole: middle temporal gyrus	TPOmid	Modular V
Calcarine fissure and surrounding cortex	CAL	Modular II	Inferior temporal gyrus	ITG	Modular IV
Cuneus	CUN	Modular II			

Modular I: the somatosensory and auditory module; Modular II: the visual module; Modular III: the attention module; Modular IV: the default-mode network (DMN) module; and Modular V: the limbic/paralimbic and subcortical systems.

Table S3. Comparisons of the global network measures among the control and ASPD groups

Parcellation	E_{glob}	E_{loc}	L_p	C_p	λ	γ	σ
L-Crad (n = 200)							
ASPD	0.39 ± 0.129	0.57 ± 0.157	2.69 ± 0.652	0.42 ± 0.121	1.16 ± 0.127	1.17 ± 0.090	1.81 ± 0.927
Control	0.33 ± 0.094	0.47 ± 0.15	3.11 ± 0.618	0.35 ± 0.112	1.18 ± 0.056	1.20 ± 0.078	2.13 ± 0.752
p value	0.03*	0.012*	0.011*	0.011*	0.072	0.019*	0.13
H-1024 (n = 1024)							
ASPD	0.30 ± 0.09	0.50 ± 0.136	3.24 ± 0.69	0.35±0.102	1.26 ± 0.08	3.11 ± 1.6	2.43 ± 1.12
Control	0.26 ± 0.08	0.45 ± 0.132	3.55 ± 0.548	0.32±0.101	1.29 ± 0.057	3.95 ± 1.82	3.04 ± 1.39
p value	0.082	0.0511	0.0503	0.0895	0.025*	0.0532	0.0578

The function networks for each participant were constructed using two parcellation methods (L-Crad and H-1024). Data are expressed as the mean ± SD. (*: $p < 0.05$)

Table S4 Comparisons of the global network measures among the control and ASPD by applying the “scrubbing” method to the pre-processed data.

Parcellation	E_{glob}	E_{loc}	L_p	C_p	λ	γ	σ
ASPD	0.42 ± 0.125	0.68 ± 0.156	2.71 ± 0.576	0.52 ± 0.345	1.56 ± 0.127	1.58 ± 0.546	1.32 ± 0.372
Control	0.38 ± 0.823	0.55 ± 0.143	2.83 ± 0.364	0.48 ± 0.310	1.62 ± 0.060	1.97 ± 0.455	1.68 ± 0.654
p value	0.023*	0.019*	0.074	0.008*	0.401	0.004*	0.002*

Data are expressed as the mean ± SD. (*: $p < 0.05$)

E_{glob}: global efficiency; E_{loc}: local efficiency; C_p: clustering coefficient; L_p: characteristic path length; γ: normalised clustering coefficient; λ: normalised characteristic path length; σ: small-worldness index.

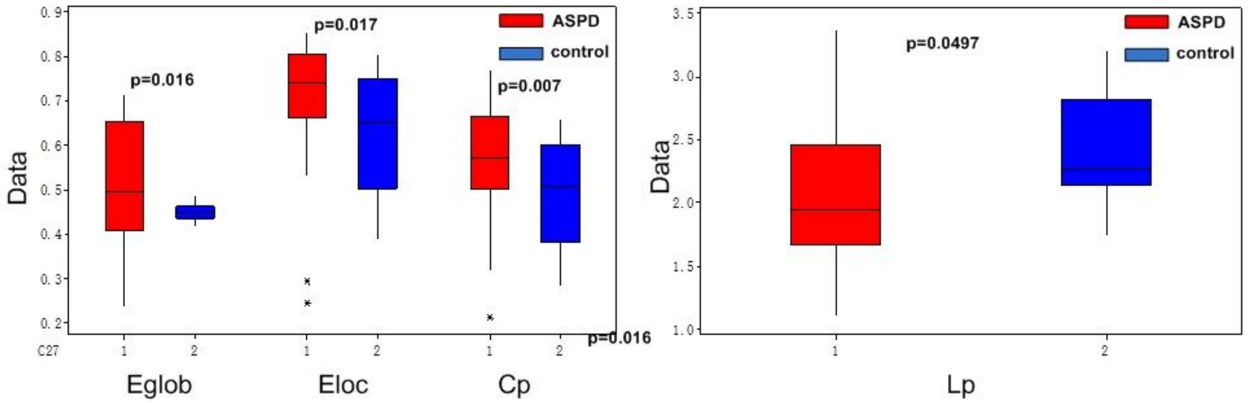

Figure S1. Group comparisons of global topological metrics in wavelet scale four. The results of the global efficiency, local efficiency, and clustering coefficient were shown in the left plot. The characteristic path length was shown in the right plot.

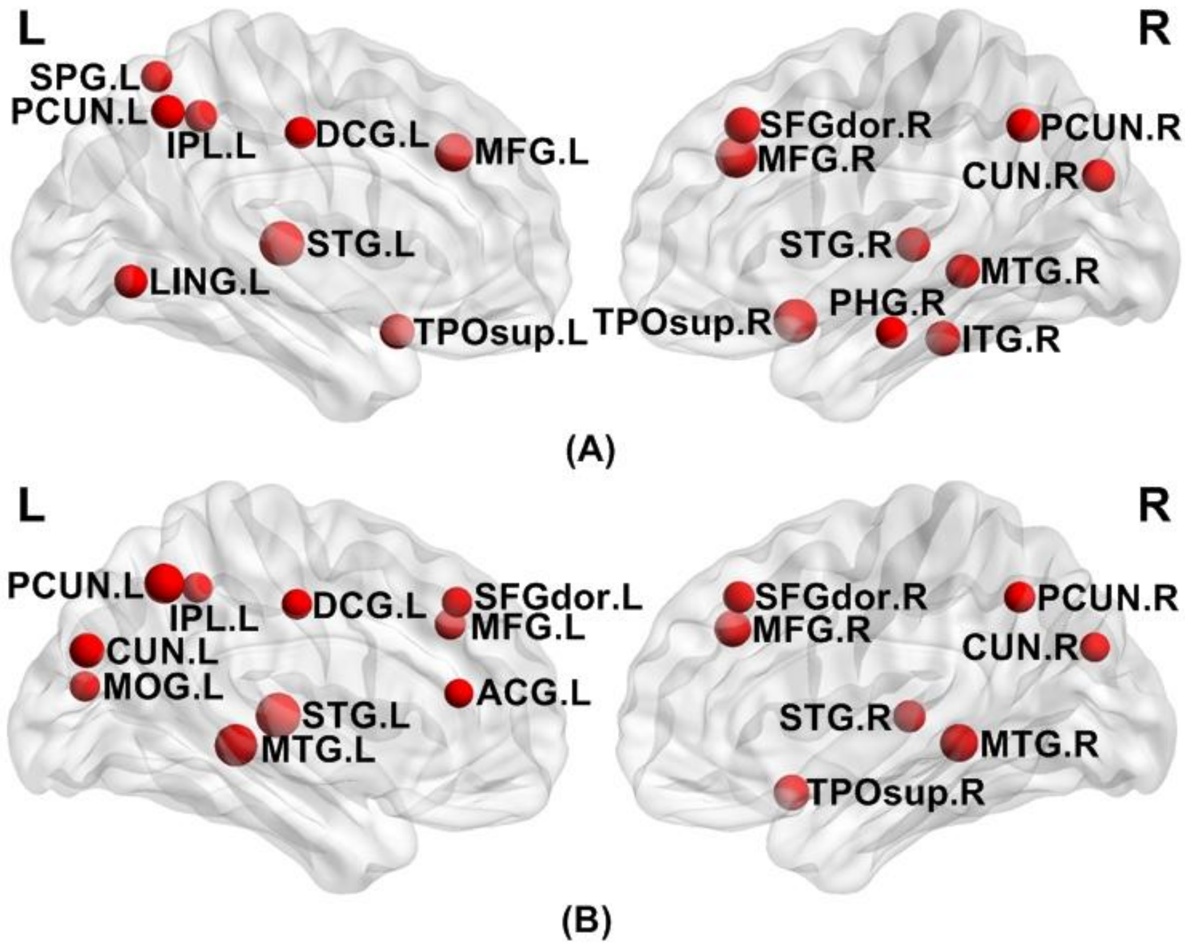

Figure S2. (A) Hubs of the functional connectome in the control group. (B) Hubs of the functional connectome in the ASPD group. SFGdor.L: left superior frontal gyrus, dorsolateral; ACG.L: left anterior cingulate and paracingulate gyri; DCG.L: left median cingulate and paracingulate gyri; SFGdor.R: right superior frontal gyrus, dorsolateral; CUN.L: left cuneus; CUN.R: right cuneus; MOG.L: left middle occipital gyrus; IPL.L: left inferior parietal, supramarginal and angular gyri; PCUN.L: left precuneus; PCUN.R: right precuneus; MFG.L: left middle frontal gyrus; MFG.R: right middle frontal gyrus; STG.L: left superior temporal gyrus; STG.R: right superior temporal gyrus; TPOsup.R: right temporal pole: superior temporal gyrus; MTG.L: left middle temporal gyrus; MTG.R: right middle temporal gyrus; PHG.R: right parahippocampal gyrus; LING.L: left lingual gyrus; SPG.L: left superior parietal gyrus; TPOsup.L: left temporal pole: superior temporal gyrus; ITG.R: right inferior temporal gyrus.

Discussion

Deficits of regional characteristics

In our paper, abnormal nodal betweenness in the frontal lobe (right Rolandic operculum, right precentral gyrus, and right medial superior frontal gyrus), parietal lobe (right superior parietal gyrus and left angular gyrus), and temporal lobe (left middle temporal pole and left middle temporal gyrus) were found in ASPD. Unlike the previous studies of structural, metabolic and functional abnormalities, the abnormal nodal betweenness in ASPD subjects was examined to evaluate the influence of a node on the information flow between the remaining nodes in a network. Nevertheless, the nodal betweenness was significantly positively correlated with blood flow and metabolism[2]. Our observations extended the current understanding of the neuroanatomical features of ASPD individuals.

Some regions with significantly decreased nodal betweenness were found in the frontal lobe (i.e. the right Rolandic operculum, the right precentral gyrus, and the right medial superior frontal gyrus), and the findings were quite consistent with the previous studies of the structural and functional abnormalities of ASPD subjects. Impulsive aggressive acts have been associated with reduced metabolism in the superior medial frontal. The precentral gyrus is part of the primary motor cortex and the deficit in precentral gyrus may increase the chance of future aggression. A recent study has reported a significantly positive correlation between a negative behavioural urgency and the grey matter volumes in the right Rolandic operculum in personality disorders with antisocial diagnosis[3]. These findings persistently suggest that the aberrant nodal betweenness in these three frontal regions might be partially involved in the violent and aggressive behaviours of ASPD individuals.

A significantly decrease in nodal betweenness centrality was observed in subjects with ASPD in the parietal lobe (the right superior parietal gyrus and left angular gyrus). Superior parietal cortex is critical for the manipulation and rearrangement of information in working memory. The superior parietal lobe is also crucial for sensorimotor integration to maintain an internal representation of the body’s state. A reduced metabolism has been found in the superior parietal cortex in aggressive patients[4], murderers[5] and individuals with impulsive personality disorders[6]. Hence, the functions of the superior parietal lobe are important for the selection and control of socially relevant behaviours. Other cognitive systems are likely to be affected if the functions of the superior parietal lobe are impaired. Moreover, violent criminals are also shown to have a reduced glucose metabolism[5] and blood flow[7] in the

angular gyrus. It has been argued that the angular gyrus was associated with the sense of responsibility for one's actions, the lack of which may contribute to immoral behaviour of ASPD individuals[8].

Besides, the temporal lobe is another major brain area associated with antisocial and aggressive behaviour[4]. In our previous study, ASPD subjects were found with a higher ReHo value in the temporal gyrus when compared with control subjects[9], and a higher ReHo value indicates a higher glucose metabolism. Hence, this is consistent with our findings of an increased nodal betweenness in the left middle temporal gyrus. Nevertheless, in this study, a significantly decreased nodal betweenness centrality was observed in left middle temporal pole of the ASPD group. Many studies have shown the temporal poles are essential for cognitive empathy[10], and the damage to the middle temporal pole may cause the development of cold-bloodedness, one of major characteristics of ASPD.

In this study, all of the ROIs are related to symptoms of depersonalization. The abnormal nodal betweenness may be connected to the high impulsivity, lack of conscience and cold-bloodedness of ASPD.

reference：

1 He, Y. *et al.* Uncovering intrinsic modular organization of spontaneous brain activity in humans. *PLoS ONE* **4**, e5226, doi:10.1371/journal.pone.0005226 (2009).

2 Liang, X., Zou, Q., He, Y. & Yang, Y. Coupling of functional connectivity and regional cerebral blood flow reveals a physiological basis for network hubs of the human brain. *PNAS* **110**, 1929-1934, doi:10.1073/pnas.1214900110 (2013).

3 Albein-Urios, N. *et al.* Negative urgency, disinhibition and reduced temporal pole gray matter characterize the comorbidity of cocaine dependence and personality disorders. *Drug Alcohol Depend.* **132**, 231-237, doi:doi:10.1016/j.drugalcdep.2013.02.008 (2013).

4 Hirono, N., Mega, M. S., Dinov, I. D., Mishkin, F. & Cummings, J. L. Left frontotemporal hypoperfusion is associated with aggression in patients with dementia. *Arch. Neurol.* **57**, 861-866, doi:doi:10.1001/archneur.57.6.861 (2000).

5 Raine, A., Buchsbaum, M. & LaCasse, L. Brain abnormalities in murderers indicated by positron emission tomography. *Biol. Psychiatry* **42**, 495-508, doi:10.1016/S0006-3223(96)00362-9 (1997).

6 Siever, L. J. *et al.* d, l-fenfluramine response in impulsive personality disorder assessed with [18F] fluorodeoxyglucose positron emission tomography. *Neuropsychopharmacology* **20**, 413-423, doi:10.1016/S0893-133X(98)00111-0 (1999).

7 Soderstrom, H. *et al.* Reduced frontotemporal perfusion in psychopathic personality. *PSYCHIAT RES:NEUROIM* **114**, 81-94, doi:10.1016/S0925-4927(02)00006-9 (2002).

8 Raine, A. & Yang, Y. Neural foundations to moral reasoning and antisocial behavior. *Soc Cogn Affect Neurosci* **1**, 203-213, doi:10.1093/scan/nsl033 (2006).

9 Tang, Y. *et al.* Altered spontaneous activity in antisocial personality disorder revealed by regional

homogeneity. *Neuroreport* **24**, 590-595, doi:10.1097/WNR.0b013e3283627993 (2013).

10 Schnell, K., Bluschke, S., Konradt, B. & Walter, H. Functional relations of empathy and mentalizing: an fMRI study on the neural basis of cognitive empathy. *Neuroimage* **54**, 1743-1754, doi:10.1016/j.neuroimage.2010.08.024 (2011).
